# Investigation of Cancer Cell Migration and Proliferation on Synthetic Extracellular Matrix Peptide Hydrogels

**DOI:** 10.3389/fbioe.2020.00773

**Published:** 2020-09-04

**Authors:** Zbigniev Balion, Emilija Sipailaite, Gabija Stasyte, Agne Vailionyte, Airina Mazetyte-Godiene, Ieva Seskeviciute, Rasa Bernotiene, Jaywant Phopase, Aiste Jekabsone

**Affiliations:** ^1^Institute of Pharmaceutical Technologies, Lithuanian University of Health Sciences, Kaunas, Lithuania; ^2^Ferentis UAB, Vilnius, Lithuania; ^3^Department of Nanoengineering, Center for Physical Sciences and Technology, Vilnius, Lithuania; ^4^Laboratory of Molecular Neurobiology, Neuroscience Institute, Lithuanian University of Health Sciences, Kaunas, Lithuania; ^5^Department of Physics, Chemistry and Biology, Linköping University, Linköping, Sweden

**Keywords:** collagen like peptide, cell adhesion peptides, glioblastoma, melanoma, migration, proliferation, focal adhesion

## Abstract

Chemical and mechanical properties of a tumor microenvironment are essential players in cancer progression, and it is important to precisely control the extracellular conditions while designing cancer *in vitro* models. The study investigates synthetic hydrogel matrices from multi-arm polyethylene glycol (PEG) functionalized with collagen-like peptide (CLP) CG(PKG)_4_(POG)_4_(DOG)_4_ alone and conjugated with either cell adhesion peptide RGD (mimicking fibronectin) or IKVAV (mimicking laminin). Human glioblastoma HROG36, rat C6 glioma cells, and A375 human melanoma cells were grown on the hydrogels and monitored for migration, proliferation, projected cell area, cell shape index, size and number, distribution of focal contacts in individual cells, and focal adhesion number. PEG-CLP-RGD induced migration of both glioma cell lines and also stimulated proliferation (assessed as metabolic activity) of HROG36 cells. Migration of C6 cells were also stimulated by PEG-CLP-IKVAV. These responses strongly correlated with the changes in adhesion and morphology parameters of individual cells – projected cell area, cell shape index, and focal contact number. Melanoma A375 cell proliferation was increased by PEG-CLP-RGD, and this was accompanied by a decrease in cell shape index. However, neither RGD nor IKVAV conjugated to PEG-CLP stimulated migratory capacity of A375 cells. Taken together, the study presents synthetic scaffolds with extracellular matrix (ECM)-mimicking peptides that allow for the exploration of the effect of ECM signaling to cancer cells.

## Introduction

The design of a realistic cell culture microenvironment is an important aspect in the development of cancer *in vitro* models. The way cancer cells sense and respond to both chemical and mechanical cues might strongly affect tumor cell invasiveness and modulate the disease progression (Papalazarou et al., 2018). Cancer cell ability to invade healthy tissue makes the difference between not very dangerous locally growing tumors and life-threatening systemic disease (Friedl and Alexander, 2011). Besides soluble and extracellular vesicle-encapsulated factors, there are two key players instructing cancer cells to migrate and proliferate: cell–cell interaction and cell–extracellular matrix (ECM) interaction. For studies into the latter, it is necessary to develop a substrate that: (i) would mimic a natural cancer cell environment, (ii) would have precisely controlled composition of signaling elements, and (iii) would support a standardized and easy-to-monitor and analyze cell culture.

The most important and best studied structural proteins of ECM are collagens, fibronectins, and laminins (Paolillo and Schinelli, 2019). Collagens characterized by a supramolecular helix structure formed from three polypeptide α-chains are the most abundant proteins of ECM and make up about one third of the total human protein mass (Shoulders and Raines, 2009). Fibronectin dimers connect ECM elements by binding to collagens and other fibronectin molecules (Oxford et al., 2019). Heterotrimeric laminins form independent networks or bind to other ECM proteins (Colognato and Yurchenco, 2000). Collagens, laminins, and fibronectins provide binding sites for cellular integrin receptors to make focal adhesions (Mostafavi-Pour et al., 2003; Berrier and Yamada, 2007; Nissinen et al., 2012). Cell–matrix adhesions, or focal adhesions, are essential for the regulation of biological processes such as cell survival, proliferation, and tumorigenesis (Berrier and Yamada, 2007). Integrins act as receptors for ECM targets by transmitting outside-in and inside-out signaling that involves over 50 proteins (Zamir et al., 1999; Hynes, 2004). By means of focal adhesions, cells relocate and receive mechanical stimuli from the environment (Ingber, 2003; Berrier and Yamada, 2007; Parsons et al., 2010). Moreover, the cell migration directly depends on the focal adhesion size (Kim and Wirtz, 2013). These interactions between cells and ECM proteins control differentiation, shape, movement, cell phenotype, and viability (Colognato and Yurchenco, 2000; Smith et al., 2018). It is established that laminin produced by keratinocytes promotes both adhesion and migration of melanocytes and melanoma cells (Chung et al., 2011). On its turn, fibronectin increases the malignancy of glioma stem-like cells modulating the differentiation, proliferation, and chemoresistance via cell adhesion signaling (Yu et al., 2018). Thus, it is evident that the presence of these proteins in the environment of cancer cells makes a difference and it is important to have at least main ECM signals for designing realistic cancer *in vitro* models. However, here lies the challenge of controlled design, scaling, and standardization of such ECM mimetics, because the production of the proteins is expensive and contains the risk of relatively high batch-to-batch variations and biocontamination.

A promising strategy in ECM engineering was introduced by the discovery of so-called cell adhesion peptides, or short amino acid sequences, that contain the minimal information required to specifically bind to a cell receptor responsible for the cell adhesion (Huettner et al., 2018). The ability of RGD sequence to promote cell attachment in a way similar to fibronectin was demonstrated for the first time by Pierschbacher and Ruoslahti (1984), and soon after this, the peptide was applied for the designing of a cell-instructing hydrogel matrix (Hern and Hubbell, 1998; Rowley and Mooney, 2002; Nemir et al., 2010; Wall et al., 2010). Peptide motif IKVAV was first presented as a sequence responsible for neuritogenic bioactivity in laminin α-chain by Nomizu et al. (1995) and subsequently applied for the functionalization of hydrogels in neural tissue engineering (Adams et al., 2005). Efforts to design a synthetic collagen with self-assembling triple helical structure were successful in the studies of O’Leary et al. (2011), who managed to achieve the formation of homogeneous collagen mimetic nanofibers. Moreover, the peptides were able to form hydrogels with properties very similar to those of natural collagen. More recently, the collagen-mimicking triple helix peptides were stabilized by physical crosslinking to the polymeric scaffolds and exploited as all-synthetic ECM for tissue engineering (Luo and Kiick, 2013; Yuan et al., 2016).

Hydrogels are among the most attractive materials for tissue engineering due to their similarity to *in vivo* cellular microenvironments (Drury and Mooney, 2003). They are usually made by translating a hydrophilic polymer solution into a 3D network structure via physical or chemical crosslinking (Tibbitt and Anseth, 2009). Synthetic hydrogels comprising such ECM signal mimicking peptides are considered as a new promising tool in cancer model development because of their well-defined composition, structural integrity and robustness, controlled charge, stiffness, porosity, nanostructure, degradability, and adhesion properties (Worthington et al., 2015).

In this study, we examine ECM-mimicking hydrogels made of self-assembling collagen-like peptide attached to eight-armed polyethylene glycol (PEG-CLP; Islam et al., 2016) functionalized with fibronectin active site motif RGD (PEG-CLP-RGD) and laminin motif IKVAV (PEG-CLP-IKVAV) as matrices for cancer *in vitro* modeling. Previously, the hydrogels have promoted the attachment and self-assembly of primary neuronal-glial cells from developing rat cerebellum to functional organoids (Balion et al., 2020). In this study, the matrices were tested with two cancerous cell lines of astrocytic origin: rat glioma cells C6 and human glioblastoma HROG36. Also, a cancerous cell line of different origin human melanoma A375 was examined for comparison. All the three cell lines were investigated for migration, proliferation, and focal adhesion formation on PEG-CLP, PEG-CLP-RGD, and PEG-CLP-IKVAV hydrogel samples.

## Materials and Methods

### Hydrogel Substrate Fabrication

Unless otherwise stated, all chemicals were purchased from Sigma-Aldrich.

The synthesis of CLPs, conjugation with multi-arm PEG, and CLPs-PEG hydrogels preparation were performed following previously described protocol (Islam et al., 2016). Peptides CLP (Cys-Gly-(Pro-Lys-Gly)_4_(Pro-Hyp-Gly)_4_(Asp-Hyp-Gly)_4_), CLP-RGD (-Arg-Gly-Asp-Ser-Pro-Gly), and CLP-IKVAV (-Ile-Lys-Val-Ala-Val-Gly) were synthesized by UAB Ferentis (Vilnius, Lithuania). Functionalized PEG-CLP hydrogels containing additional motifs responsible for cell adhesion to the ECM (RGD) or laminin (IKVAV) mimetic motifs were prepared in the same manner as PEG-CLP. Briefly, peptides were conjugated to 40 kDa 8-arm PEG-maleimide (JenKem, TX, United States). The composition of peptides and conjugation of CLP, CLP-RGD, and CLP-IKVAV with 8-arm PEG-maleimide were characterized using ^1^H NMR on a Bruker Ascend 400 MHz spectrometer at room temperature. Briefly, 2% solutions of CLP, CLP-RGD, CLP-IKVAV, PEG-CLP, PEG-CLP-RGD, and PEG-CLP-IKVAV were prepared in deuterium oxide (D_2_O). The resonance of deuterated solvent (D_2_O, δ = 4.79) was used as the internal standard.

Further, 12% (w/w) PEG-peptide aqueous solution was crosslinked with 4-(4,6-dimethoxy-1,3,5-triazin-2-yl)-4-methylmorpholinium chloride (DMTMM) at RT (Haagdorens et al., 2019). Due to the hydrophobic nature of the CLP-IKVAV peptide, 10% (w/w) PEG-CLP-IKVAV solution was used. Amine molar ratio of PEG-peptide-NH_2_ to DMTMM was 1:2. After thorough mixing, final solution containing 8.5 ± 0.2% (AVG ± STDEV, w/w) PEG-CLP, 8.5 ± 0.2% (w/w) PEG-CLP-RGD or 7.2 ± 0.2% (w/w) PEG-CLP-IKVAV was cast between two glass plates to form a flat 500 μm thickness sheet. The hydrogels were left to cure overnight in 100% humidity at RT. The molar concentration of peptides in the hydrogels were: 1.17 ± 0.10 mM of CLP in PEG-CLP, 1.17 ± 0.02 mM CLP-RGD in PEG-CLP-RGD, and 0.99 ± 0.02 mM of CLP-IKVAV in PEG-CLP-IKVAV. After crosslinking, 6 mm hydrogel disks were cut from the fabricated sheets using a threphine. Prior to use, hydrogels were kept refrigerated inside sterile vials filled with phosphate buffer solution containing 1% (v/v) chloroform to maintain sterility (Islam et al., 2018).

#### 2D Cultures

HROG36 (RRID: CVCL_4U49), C6 (RRID:CVCL_0194), and A375 (RRID: CVCL_0132) cell lines were purchased from Cell Lines Service GmbH (Germany).

The cells were cultured in the specific medium (DMEM/Ham’s F12 1:1 for HROG36, DMEM for C6 and RPMI for A375) and supplemented with 10% 0.22 μm pore filter-sterilized fetal bovine serum (FBS) and 1% penicillin-streptomycin at 37°C and 5% CO_2_. Cells were grown in T75 flasks (15–20 mL of medium) until 75-95% confluency, then detached by 0.25% Trypsin-EDTA solution and used for spheroid formation and proliferation evaluation.

#### Spheroid Formation

The “hanging drop” method was used to form spheroids of a similar shape and size.

For HROG36 and C6 cells, 5 μL (26,000 cells) drops of glioblastoma cell suspension were placed on the inside of the sterile tissue culture dish cover. Then the culture dish cover was turned around and placed on the respective cell dish containing 10 mL Phosphate-buffered saline (PBS). Spheroids were grown at 37°C temperature for about 24 h and after that were transferred into the 24 well plate, the bottom of which was covered with 1% agarose. Transferred spheroids were grown in the cell culture medium at 37°C temperature. Spheroids were fully formed after 5 days; the diameter of the spheroids for C6 cells was 113 ± 13 μm, and for HROG36 – 198 ± 59 μm.

For A375 spheroid formation, a slightly different procedure was applied due to different proliferation and spheroid formation capacities of the cells. Both glioma cell lines form connections faster and can join to integral spheroids at lower cell numbers compared to A375 melanoma cells. However, A375 cannot survive in hanging drops in larger amounts because of its very high proliferation rate and restricted amount of nutrients in small medium volume. Thus, seed-spheroids were initially formed by spotting 25 μL drops (500 cells) of the cell suspension onto the inner surface of the lid of a sterile non-adhesive cell culture dish. The lid was placed on the respective cell culture dish containing 10 mL PBS. After 10 days, the seed spheroids were gently transferred on agarose for further growth until the formation of integral shapes retaining spheroids of 473 ± 96 μm in diameter.

### Cell Migration Assessment

The formed spheroids were pipetted onto PEG-CLP, PEG-CLP-RGD, and PEG-CLP-IKVAV hydrogels, and incubated for 24 h. The brightfield phase contrast images of spheroids and cells migrated on hydrogels were taken by microscope Leica DMi1 and analyzed by Image J freeware. The migration was assessed by measuring the size of the area covered by cells around the spheroid (excluding the area covered by the spheroid itself), and by counting the number of cells in the area.

### Cell Proliferation Evaluation

Cell proliferation was assessed by evaluating the metabolic activity of the cells by means of PrestoBlue^TM^ Cell Viability Reagent (Thermo Fisher Scientific). This is a resazurin-based, membrane permeable solution that upon reduction forms resorufin, a compound that is red and highly fluorescent. The rate of conversion corresponds to the rate of metabolic activity of the cells and can be applied for assessment of cell proliferation as described in Czekanska (2011). The procedure was performed according to the protocol provided by the manufacturer. For this, each cell type was seeded on hydrogel disks at a density of 3000 cells/well of a 96 well plate. Cell proliferation was assessed after 24 and 48 h. 10 μL of PrestoBlue^TM^ reagent was added to each well of 96-well plate containing 90 μL of cell culture medium and incubated for 40 min at 37°C in a cell culture incubator, protected from direct light. After incubation, the fluorescence of resorufin was detected using a multimode plate reader Infinite M Plex (Tecan Austria, Salzburg, Austria) at excitation and emission wavelengths of 560 and 590 nm, respectively. The results were expressed as means ± standard deviation of relative fluorescent units.

### Immunocytochemistry

For the evaluation of focal adhesions, the cells were fixed with 4% paraformaldehyde in PBS for 5 min, permeabilized with 0.1% Triton X-100 for 5 min, and then incubated in 1% BSA in PBS for 30 min to block non-specific protein–protein interactions. The cells were then incubated with the primary vinculin-binding antibody (ab18058, RRID:AB_444215, diluted 1:200 making final concentration 3 μg/mL) for 2 h at room temperature. The secondary antibody ab150113 Alexa Fluor^®^ 488 goat anti-mouse IgG (H + L, RRID:AB_2576208) used at a concentration of 2 μg/mL was applied for 45 min together with Texas Red^TM^-X Phalloidin (Invitrogen, T7471, diluted 1:40 in 1% BSA, final concentration 5 units/mL, or 165 nM) for F-actin staining and 1.43 μM DAPI for nuclear visualization. The cell images were taken by fluorescent microscope Zeiss Axio Observer.Z1 (Carl Zeiss, Jena, Germany). Size distribution of individual focal contacts was plotted for three individual cells of each cell line on each hydrogel substrate. Gausian 4 parameter equation curves were fitted to the data by SigmaPlot software. Cell shape index was calculated as described in Cornhill et al. (1980), applying the equation:

$$\mathrm{Shape}\,\mathrm{index}=4\,\pi \,A/P^{2}$$

where *A* is the cell area and *P* is the cell perimeter. Cell perimeter and cell area were calculated by means of ImageJ software. The number of focal adhesions were also evaluated by ImageJ as vinculin and actin colocalization points per cell.

### Statistical Analysis

The quantitative results are presented as mean ± standard deviation of 3–12 replicates. The statistical data analysis was performed by applying the ANOVA with LSD *post hoc* test. Differences were considered statistically significant when *p* < 0.05. The data were processed using Microsoft Office Excel 2010 (Microsoft) and SPSS 20 (IBM) software. Correlations were analyzed by means of Microsoft Office Excel 2010 (Microsoft) software using Correlation function.

## Results

### PEG-Peptide Synthesis and Characterization

**FIGURE 1:**
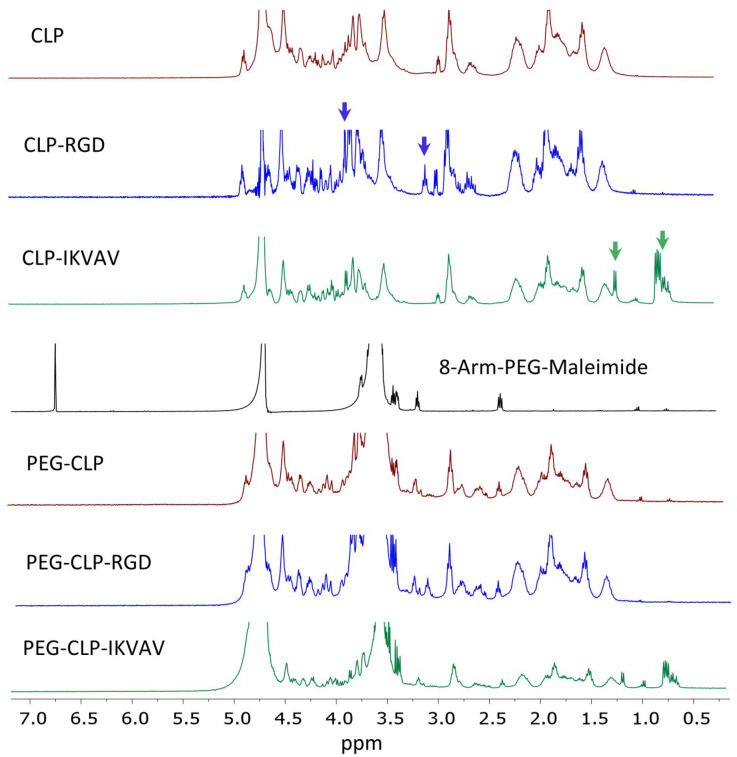
NMR spectra of CLP, CLP-RGD, CLP-IKVAV, 8-arm-PEG (40kDa) and PEG-CLP derivatives. Blue arrows mark arginine at δ 3.10 ppm and serine peaks at δ 3.90 ppm of CLP-RGD peptide. Green arrows show valine at δ 0.75 ppm, isoleucine at δ 0.82 ppm, and alanine peaks at δ 1.22 ppm of CLP-IKVAV peptide. The maleimide peaks at δ 6.5–7.0 ppm disappeared after conjugation with CLPs peptides.

The peptide synthesis and peptide conjugation to PEG-maleimide was confirmed by ^1^H NMR spectroscopy. The ^1^H NMR spectra of CLP was similar to earlier published spectra (Islam et al., 2016). The CLP-RGD spectra had an additional arginine peak at δ 3.10 ppm and serine peak at δ 3.90 ppm (marked by blue arrows in Figure 1) from RGD motif (Balion et al., 2020). The CLP-IKVAV had an apparent valine peak at δ 0.75 ppm, isoleucine peak at δ 0.82 ppm, and alanine peak at δ 1.22 ppm (marked by green arrows in Figure 1) from additional IKVAV motif. The quantitative conjugation of CLP, CLP-RGD, and CLP-IKVAV peptides with 8-arm-PEG maleimide was confirmed by the disappearance of the maleimide peak at δ 6.5–7.0 ppm after conjugation of peptides to PEG template. The nominal surface concentration of the peptides estimated from molar concentration of each peptide was 1 pmol/cm^2^, the values close to those previously reported by Hern and Hubbell, 1998.

### Cancer Cell Migration on PEG-CLP Hydrogels

**FIGURE 2:**
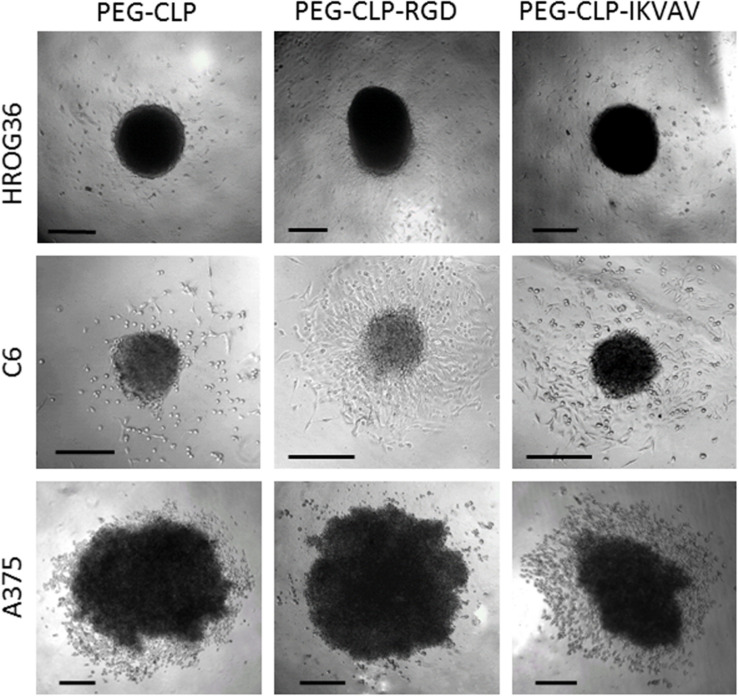
Representative images of HROG36, C6, and A375 cell spheroid cultures on PEG-CLP, PEG-CLP-RGD, and PEG-CLP-IKVAV hydrogels after 24 h from spheroid positioning. Scale bar is 100 μm.

**FIGURE 3:**
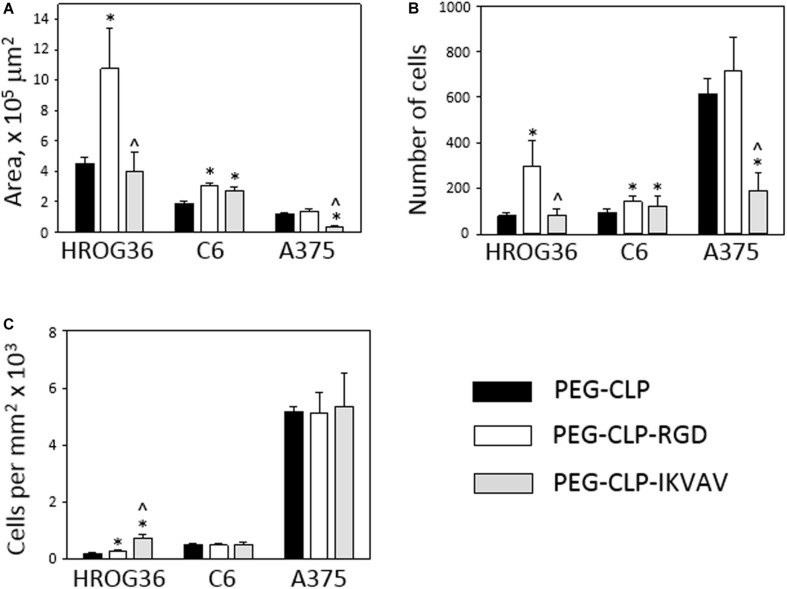
Migration of HROG36, C6, and A375 cells on PEG-CLP, PEG-CLP-RGD, and PEG-CLP-IKVAV hydrogels. The cell migration was evaluated by measuring the area of cell spreading around the spheroid in 24 h **(A)**, by counting cells in the area **(B)**, and by indicating cell number per mm^2^ **(C)**. The experiments were performed in three independent experimental sets, each of three replicates. *Indicates statistically significant difference compared with the samples of the same cell type grown on PEG-CLP hydrogels, ^∧^ – with samples on PEG-CLP-RGD hydrogels, ANOVA with *post hoc* LSD test, *p* < 0.05.

To evaluate HROG36, C6, and A375 cell migration induced by different cell adhesion peptides, the spheroids were formed and placed on PEG-CLP, PEG-CLP-RGD, and PEG-CLP-IKVAV hydrogels. After 24 h, the area occupied by cells was measured and the number of cells around the spheroids was counted. The representative images of the spheroid-cultures on the hydrogels after 24 h are presented in Figure 2. The largest migration areas were found in human glioblastoma HROG36 samples, and the smallest areas in melanoma A375 samples (Figures 2, 3A). The introduction of RGD signals to the PEG-CLP core dramatically increased the spreading area of HROG36 glioblastoma cells; the cell spreading area on PEG-CLP-RGD was 2.38 times bigger compared to that on PEG-CLP hydrogels (Figure 3A). On the contrary, laminin motif IKVAV did not significantly influence HROG36 cell migration and the spreading area on PEG-CLP-IKVAV remained similar to PEG-CLP. Migration of rat glioma C6 line cells was similarly stimulated both by fibronectin active sequence RGD and IKVAV from laminin. The average spreading area was by 1.68 times bigger on PEG-CLP-RGD and 1.45 times bigger on PEG-CLP-IKVAV compared to the area on PEG-CLP. Human melanoma cells A375 migrated similarly on both PEG-CLP and PEG-CLP-RGD. However, introduction of the IKVAV motif significantly blocked the melanoma cell migratory capacity. The cell spreading area on PEG-CLP-IKVAV was 79% smaller than on PEG-CLP (Figure 3A).

Counting cells in the area of spread around the spheroids after 24 h revealed the highest numbers in A375 melanoma samples, approximately 2–3 times lower in human glioblastoma HROG36 samples and about 2 times lower in rat glioma C6 samples. Similarly to the spreading area results, the number of glioblastoma HROG36 cells was 3.67 times bigger on PEG-CLP-RGD than on PEG-CLP, and IKVAV sequence did not significantly change cell number around the spheroid after 24 h (Figure 3B). The number of spread C6 cells was 1.56 times higher on PEG-CLP-RGD compared to the samples cultivated on PEG-CLP hydrogels. Laminin motif IKVAV significantly decreased cell number around the A375 spheroids; it was 69% smaller compared to that on PEG-CLP.

The calculation of cell number per unit area revealed more than 10 times higher values in A375 samples compared to both glioma cell lines HROG36 and C6 (Figure 3C). However, there was no significant difference in cell per unit area number between PEG-CLP, PEG-CLP-RGD, and PEG-CLP-IKVAV hydrogels in both A375 and C6 cell cultures. In contrast, the number of HROG36 cells per areal unit slightly yet significantly increased on PEG-CLP-RGD compared to PEG-CLP, and markedly increased on PEG-CLP-IKVAV compared to both PEG-CLP and PEG-CLP-RGD samples.

To summarize, human glioblastoma HROG36 cell cultures make the largest spreading area of all investigated cancer cell types, but the highest cell numbers around the spheroid and the highest cell per unit area values were found in human melanoma A375 samples. Both HROG36 and C6 cell migration was stimulated by RGD peptide, but C6 cells were similarly stimulated by IKVAV. On the contrary, in A375 case, IKVAV peptide suppressed both spreading area and cell number. The difference in cell per unit area values between different hydrogels was found only in HROG36 cell cultures.

### Cancer Cell Proliferation on PEG-CLP Hydrogels

**FIGURE 4:**
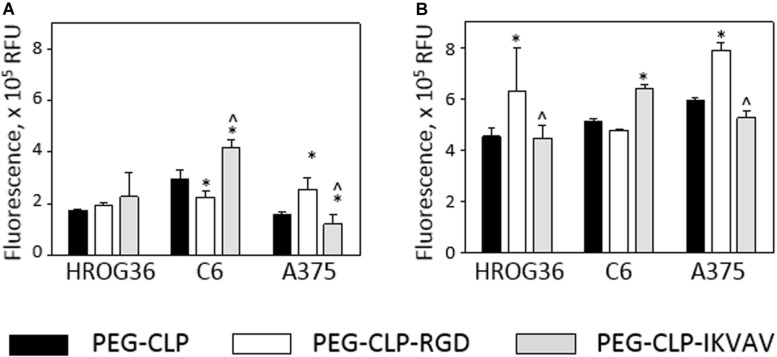
Metabolic activity of HROG36, C6, and A375 cells on PEG-CLP, PEG-CLP-RGD, and PEG-CLP-IKVAV hydrogels after 24 h **(A)** and 48 h **(B)**. The metabolic activity of the cells corresponding to the proliferation intensity was assessed by Presto Blue^TM^ assay in three independent experimental sets, each of three replicates. RFU, relative fluorescence units. *Indicates a statistically significant difference compared with the samples of the same cell type grown on CLP-only containing hydrogels, ^∧^-with samples on PEG-CLP-RGD hydrogels, ANOVA with *post hoc* LSD test, *p* < 0.05.

Changes in cell spreading area and especially in cell number around the spheroids might be induced by the adhesion peptide influence on cell proliferation. Therefore, next in the study, metabolic activity Presto Blue^TM^ assay was applied to estimate cell proliferation. After 24 h, there were no significant proliferation differences between HROG36 samples on different hydrogel types (Figure 4A). It is not surprising, taking into account that the reported doubling time of the cells is 35–40 h (Mullins et al., 2013). When the evaluation time was prolonged to 48 h, the proliferation rate of HROG36 cells on PEG-CLP-RGD hydrogels was 2.25 times higher than on PEG-CLP (Figure 4B). For other cells evaluated in the study, the doubling time is shorter: 24 h for the C6 cells and 16 h for A375 cells, respectively (Benda et al., 1968; Benga, 2001). Indeed, in the samples of the latter cells with a shorter doubling time, the differences in proliferation rate on different hydrogels were already visible after 24 h. RGD significantly blocked the proliferation of C6 cells compared to PEG-CLP samples (Figure 4A). The average level of cell proliferation in C6 samples on PEG-CLP-RGD hydrogels were 33% lower than that on PEG-CLP hydrogels. In contrast, introduction of IKVAV peptide to the PEG-CLP core increased C6 proliferation rate by 1.3 times. RGD peptide increased the proliferation rate of A375 cells; on PEG-CLP-RGD hydrogels, it was 1.61 time higher compared to the PEG-CLP samples (Figure 4A). Evaluation of the proliferation after 48h confirmed the proliferation stimulating effects of RGD for C6 and A375 cells (Figure 4B). However, the extent of the effects was not so significant as after 24 h; the increase in the proliferation of the C6 cells compared to PEG-CLP hydrogel samples was 28%, and that of A375 cells was 33%.

Proliferation assessment results indicate that both human glioblastoma HROG63 and melanoma A375 cell proliferation is stimulated by RGD signaling, but the proliferation of C6 cells is stimulated by the IKVAV.

### Evaluation of Focal Adhesions on PEG-CLP Hydrogels

**FIGURE 5:**
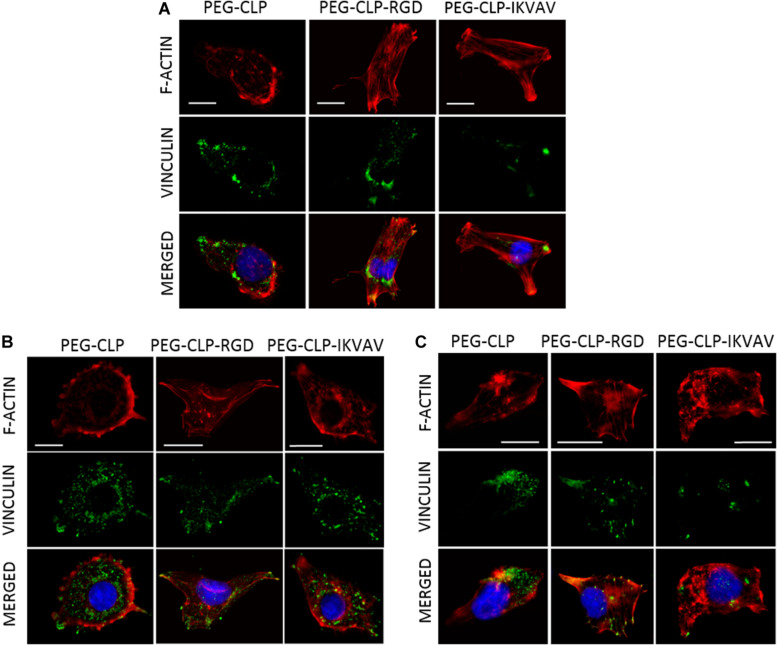
Representative images of focal adhesions formed by HROG36 **(A)**, C6 **(B)**, and A375 **(C)** cells on CLP, CLP-RGD, and CLP-IKVAV hydrogels. The cells were immunostained for actin (red signal) and vinculin (green). Nuclei were visualized blue by DAPI. Scale bar is 10 μm.

**FIGURE 6:**
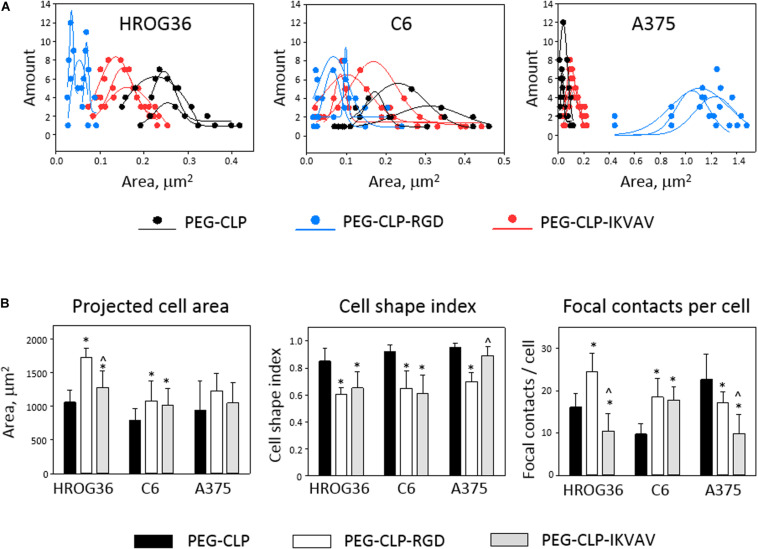
Size distribution of individual focal contacts **(A)**, projected cell area, cell shape index, and number of focal contacts **(B)** of HROG36, C6, and A375 cells on PEG-CLP, PEG-CLP-RGD, and PEG-CLP-IKVAV hydrogels. The cells were immunostained for F-actin (red signal) and vinculin (green). Nuclei were visualized blue by DAPI. Size distribution of focal contacts of three cells on each hydrogel was plotted together with approximate distribution curve. Projected cell area and perimeter for cell shape index calculation were measured by ImageJ software. Focal adhesion number per cell was calculated according to the number of vinculin-actin colocalization points. The data are presented as AVG ± SDEV of 5–7 cells from three of each hydrogel sample. *Indicates statistically significant difference compared with the samples of the same cell type grown on PEG-CLP, ∧ – compared with samples on PEG-CLP-RGD. ANOVA with *post hoc* LSD test, *p* < 0.05.

Both migration and proliferation of cancer cells can be controlled by focal adhesions to the ECM proteins (Provenzano and Keely, 2011; Nagano et al., 2012). To test if there are correlations between the effects of different hydrogels on cell migration, proliferation, and adhesion, we have evaluated the number of focal adhesions formed by cancer cells on PEG-CLP, PEG-CLP-RGD, and PEG-CLP-IKVAV hydrogels. The representative images of the cells after immunostaining for the cytoskeleton protein actin and focal adhesion forming protein vinculin is presented in Figure 5. There were many round- or oval-shaped cells on PEG-CLP hydrogels in HROG36 and C6 cell cultures with little actin fibers and small amoeboid lamellipodia. In A375 samples on PEG-CLP hydrogels, the cells were mostly rod-shaped and had parallel organization of actin filaments (Figure 5C). The presence of externally extended focal contacts with parallel and unparallel actin stress fibers were best visible on PEG-CLP-RGD hydrogels in all investigated cell types. This was also confirmed by the significantly lower cell shape index on this hydrogel substrate in all investigated cell cultures (Figure 6B, Cell shape index). Projected cell area on PEG-CLP-RGD hydrogels compared to PEG-CLP, however, was significantly higher only in both glioma cell cultures (Figure 6B, Projected cell area). Morphology of both types of glioma cells was also well defined on PEG-CLP-IKVAV (Figures 5A,B). The cells were elongated and had contact points at the ends. Projected cell area for both HROG36 and C6 was significantly higher, and cell shape index significantly lower on PEG-CLP-IKVAV hydrogel compared to PEG-CLP samples (Figure 6B). However, for HROG36 the average projected cell area on PEG-CLP-IKVAV was significantly lower compared to that on PEG-CLP-RGD. There was no significant difference in projected cell area between tested hydrogels revealed in A375 experimental group, however, the morphology of actin filaments was visibly less organized on PEG-CLP-IKVAV compared to PEG-CLP (Figures 5C, 6B).

Examination of the size distribution of focal contacts in individual cells revealed the same tendency for HROG36 and C6 cells to make smaller amounts and larger area size contacts on PEG-CLP, increase in number and decrease in contact size on PEG-CLP-IKVAV, and further increase in number/decrease in size on PEG-CLP-RGD (Figure 6A). The distribution had completely different characteristics for A375 melanoma cells that made much larger contacts on PEG-CLP-RGD than on PEG-CLP or PEG-CLP-IKVAV.

In all investigated cell types, the number of focal adhesions was similar, ranging from about 10 to 25 in average (Figure 6B, Focal contacts per cell). The highest numbers (about 20–30) of focal adhesions were found in HROG36 glioblastoma cells on PEG-CLP-RGD hydrogels and in A375 melanoma cells on PEG-CLP hydrogels. The next slightly lower level of focal contacts (from 13 to 20) was induced to C6 cells by both PEG-CLP-RGD and PEG-CLP-IKVAV and to A375 cells by PEG-CLP-RGD. The lowest amount of focal contacts (from 5 to 15) was found in PEG-CLP-IKVAV cultures of HROG36 and A375 cells, and in PEG-CLP cultures of C6 cells. The differences in focal adhesions within the same cell cultures on different hydrogels were as follows. For HROG36 cell line, the amount of focal contacts per cell was 1.51 times bigger on PEG-CLP-RGD than on PEG-CLP, and 2.33 times bigger than on PEG-CLP-IKVAV. Moreover, the number of focal contacts per cell was 1.54 times bigger on PEG-CLP than on PEG-CLP-IKVAV. The amount of focal contacts per cell in C6 cultures was 1.9 times bigger on PEG-CLP-RGD than on PEG-CLP, and 1.82 times bigger on PEG-CLP-IKVAV than on PEG-CLP. The number of focal contacts per cell of A-375 cells was 1.32 times bigger on PEG-CLP than on PEG-CLP-RGD, and 2.28 times bigger than on PEG-CLP-IKVAV.

Correlations between cell behavior (migration/proliferation) and individual cell morphology/adhesion parameters are presented in Table 1. There were strong positive correlations found between the number of focal contacts per cell and the area covered by cells from the spheroids (*r* values between 0.81 and 0.98), and number of focal contacts and total cell number (*r* between 0.91 and 1.00) in all investigated cell types. Both glioma cell lines also demonstrated strong positive correlations between projected individual cell area and area all cells covered due to migration (*r* values 0.92 and 1.00 for HROG36 and C6 cells, respectively), and between projected cell area and cell number (0.95 and 1.00 for HROG36 and C6, respectively). Only HROG36 cells showed strong positive correlations between proliferation rate estimated by metabolic activity and number of focal contacts (*r* = 0.91). This correlation was observed only when metabolic activity was assessed after 48 h. Also, in both human cancer cell line HROG36 and A375 cultures, there was strong positive correlation between projected cell area and metabolic activity/proliferation and projected cell area. For HROG36, this correlation was only when proliferation was monitored after 48 h (*r* = 0.95), but for A375 the same *r* = 0.80 correlation was established for both 24 and 48 h proliferation data sets. Both glioma cell lines showed negative correlations between cell shape index values and migration parameters. The correlation was strong for C6 cells (−0.93 and −0.95 for total cell spread area and cell number, respectively), and mild for HROG36 (−0.59 and −0.66). Also, strong negative correlations were found between the number of focal contacts and cell per unit area in C6 (*r* = −0.96) and A375 (*r* = −0.82) samples. In addition, a strong negative correlation was found between cell per unit area and projected cell area in C6 cell cultures (*r* = −0.99). There were no correlations between metabolic activity/proliferation and focal adhesion/morphology parameters in C6 cell line experimental group.

**TABLE 1:** Correlations between cell adhesion/morphology parameters and cell migration/proliferation.

Cell type	Focal adhesion/morphology parameter (Figure 6B)	Area covered by cells (Figure 3A)	Cell number (Figure 3B)	Cell per unit area (Figure 3C)	Metabolic activity/proliferation in 24 h (Figure 4A)	Metabolic activity/proliferation in 48 h (Figure 4A)
HROG36	Number of focal contacts per cell	0.94**	0.91**	−0.71*	–0.53	0.91**
	Cell shape index	−0.59*	−0.66*	–0.47	−0.67*	−0.65*
	Projected cell area	0.92**	0.95**	–0.04	0.19	0.95**
C6	Number of focal contacts per cell	0.98**	0.99**	−0.96**	–0.03	0.27
	Cell shape index	−0.93**	−0.95**	0.90**	–0.16	–0.45
	Projected cell area	1.00**	1.00**	−0.99**	–0.17	0.14
A375	Number of focal contacts per cell	0.81**	0.81**	−0.82**	0.34	0.34
	Cell shape index	–0.46	–0.46	0.44	−0.88**	−0.88**
	Projected cell area	0.33	0.33	–0.30	0.80**	0.80**

The numbers in the table are correlation coefficient *r* for the two parameters indicated in the upper line and first left column of the table. Strong correlation are indicated by **, and mild correlation by *.

To summarize, there were more similarities between responses of both glioma cell lines to different hydrogels than between any of the glioma lines and A375 melanoma cells. However, there were also several differences found between HROG36 and C6 cell behavior, such as response of proliferation assessed as increase in metabolic activity.

## Discussion

In this study we have investigated the interaction between synthetic PEG-CLP hydrogels, alone and with conjugated cell adhesion peptides RGD or IKVAV, and three cancerous cell lines: human glioblastoma HROG36, rat glioma C6, and human melanoma A375. The background for this research were previous studies on PEG-CLP hydrogels as human corneal implants (Islam et al., 2016) and research performed on primary cerebellar cells grown on PEG-CLP and PEG-CLP-RGD (Balion et al., 2020). In the above-mentioned research, the material was characterized for mechanical properties, circular dichroism spectra, and surface topography (Islam et al., 2016; Balion et al., 2020). It was found that the values of storage modulus G’ modulus of the materials were between 0.02 and 0.07 MPa, and the values of elastic (Young) E modulus were between 0.06 and 0.180 MPa, respectively. Both the shear storage and elastic moduli were lower for hydrogels with an additional peptide group attached to PEG-CLP, most likely because of the lower level of triple helical assemblies, as indicated by circular dichroism spectroscopy. The Young modulus values of the hydrogels are found to be the lowest in skin tissue; the latter vary from 0.0001 to 57 MPa, depending on the place on the body, age, and assessment conditions (Joodaki and Panzer, 2018). Regarding the mechanical properties of brain tissue, the Young modulus values reported for gray and white matter are 1.389 ± 0.289 kPa and 1.895 ± 0.592 kPa, respectively (Budday et al., 2015). Thus, the hydrogels of the current study have higher stiffness compared to the natural brain.

A nanometer-scale structure of hydrogel surfaces evaluated by atomic force microscopy (AFM) revealed relatively smooth and densely packed hydrogel surfaces with the surface roughness values measured as root mean square (RMS) between 2.7 ± 1.6 and 4.6 ± 2.8 nm. The data indicated the elastomechanical properties of the material is within the range of soft biological tissue (McKee et al., 2011), and it has the bioactive capacity to organize primary cerebellar cells to functional interconnecting organoids (Balion et al., 2020). Therefore, the idea of the study was to test whether PEG-CLP-based hydrogels might serve as an ECM mimetic to cancerous cell lines. Rat glioma C6 line was chosen as representative of brain astrocytic cancer originated from rats – the same species as cerebellar cells investigated in a previous study. HROG36 cells were selected as representative of highly invasive human glioblastoma, and human melanoma A375 cells were chosen as the other, non-glial human cancerous cell line. One of the reasons to investigate all three cell types under the same conditions was to find out if the cells respond to the peptide clues because such signals are similar to those that melanoma and glioblastoma cells might receive when growing *in vivo*. As the introduction of additional peptide motifs does not make substantial changes in hydrogel mechanical properties, the differences in cell responses between hydrogels should be attributed to the peptide signaling.

The findings of this study support the hypothesis of instructing cells with different ECM peptides. First of all, each cell line behaved in a different manner to hydrogels. In addition, hydrogels of different peptide composition induced distinct responses from the same cell type. Altogether, this suggests that PEG-CLP, PEG-CLP-RGD, and PEG-CLP-IKVAV as matrices provide a bioinstructing ECM-mimicking microenvironment for the cells. Of course, steps have to be taken to investigate the influence of the density of each peptide and their combination, to introduce other ECM molecules such as hyaluronic acid and proteoglycan signaling, and to elaborate their densities, to shape the matrix creating tissue-like structures like capillaries and nerves with the respective topography and biochemistry. Taking into account that PEG-CLP hydrogel is shape-retaining and can be precisely molded at a fine microscale level (Haagdorens et al., 2019), such organ-mimicking micro-shaping might be introduced for further microenvironmental tuning.

Regarding cellular responses on the hydrogels, the study revealed that both cell lines of astrocytic origin HROG36 and C6, as expected, had more same or similar correlations between hydrogel-induced changes of adhesion/morphology parameters and migration/proliferation capacity readouts compared to melanoma A375 cell line. Both C6 and HROG36 migrated better on PEG-CLP-RGD, and migration efficiency correlated to the number of focal contacts, projected cell area, and decrease in cell shape index. However, only human glioblastoma HROG36 cells demonstrated positive correlations between all three parameters and proliferations. Relatively rapidly proliferating rat C6 glioma cells are considered as a golden standard for complementary *in vitro* and *in vivo* glioblastoma modeling (Grobben et al., 2002). The cells share some common features with human glioblastoma, and for this reason are often used as a convenient model, allowing for the combination of *in vitro* and *in vivo* studies, because C6 gliomas can be implanted to laboratory rats and form tumors (Gunnersen et al., 2000; Gieryng et al., 2017). The current results indicate that C6-based models might have biological responses that differ from the responses of human glioblastoma, thus, the responses obtained on C6-tumors should be tested on at least one human glioblastoma cell line.

Proliferation of both human cancer cell lines HROG36 and A375 was markedly stimulated by PEG-CLP-RGD. The fact it was not revealed in metabolic activity assessment after 24 h on hydrogels might be explained by the fact that average doubling time for HROG36 is from 35 to 40 h (Mullins et al., 2013), thus, 24 h of incubation could be not enough time to reveal the response. A375 cells demonstrated very similar levels of RGD-induced stimulation of proliferation after 24 and 48 h. The cells have the shortest average doubling time (16–20 h), Benga (2001) compared to other cells investigated in the study, thus, the effect of PEG-CLP-conjugated RGD peptide could be revealed both in 24 and 48 h time periods. On the contrary, proliferation of rat glioma C6 cells was not affected by any of the investigated hydrogel matrices, and the doubling rate of those cells (about 16 h), Benda et al. (1968) could not influence this result.

Different proliferation rates of the cells could also interfere with migration results. For HROG36, this would hardly be the case; as we have examined migration for 24 h, the time is far too short for proliferation of these cells to make a noticeable influence. However, this could happen in C6 and A375 experimental groups because the doubling time of those cells is shorter than 24 h. Assessment of proliferation by metabolic activity assay revealed there was no influence of the hydrogels to C6 proliferation. Thus, both C6 and HROG36 cells have spread from the spheroid mostly because of migration. Therefore, both area covered by cells, total cell number, and cells per mm^2^ count for both glioma cells under these conditions might be assumed as parameters indicating cell migration intensity. Note that only HROG36 cells revealed differences in cells per mm^2^ between the investigated hydrogel samples, and the highest impact on this factor was made by PEG-CLP-IKVAV. An increase in cells per unit area indicates that cells are prone to migrate onto the matrix and the subsequent attachment to it slows them down and forces them to accumulate. Migration of HROG36 cells was not significantly stimulated by PEG-CLP-IKVAV compared to PEG-CLP, however, two of the three adhesion and morphology parameters (projected cell area and cell shape index) of those cells on PEG-CLP-IKVAV were similar to those on CLP-PEG-RGD, the matrix promoting the highest level of migration. This indicates a different regulatory capacity between RGD and IKVAV promoted adhesion on HROG36 glioblastoma. Although most integrins can recognize several adhesion motifs, the signal they transmit from the contact to the cell might vary (Takada et al., 2007). Even after binding the same motif, the integrins provide distinct responses depending on the RGD-containing ECM protein (vitronectin, fibronectin, fibrinogen, or other) (Kapp et al., 2017). Also, cells, including cancer cells, might have different expressions of site-specific integrins (Frith et al., 2012; Nieberler et al., 2017) and thus, respond in a different manner to these stimuli.

In contrast to the glioma cells, A375 melanoma cells were not stimulated for migration by PEG-CLP-RGD. The migration was even inhibited by PEG-CLP-IKVAV compared to PEG-CLP samples. However, these cells responded to PEG-CLP-RGD by a significant increase in proliferation rate. These data also supports the hypothesis that different cells might have distinct intracellular signaling pathways activated in response to the same ECM stimuli.

Glioblastomas are characterized by their extreme aggressiveness (Hanif et al., 2017) which is in part attributed to binding and responding to the RGD signaling via integrin β8 (Tchaicha et al., 2011). Glioblastoma cells themselves express fibronectin, and this is responsible for their invasiveness (Serres et al., 2014). Fibronectin increases glioma stem-like cell differentiation, proliferation, and chemoresistance via cell adhesion signaling (Yu et al., 2018). Thus, the results of our study are in line with the data of other researchers and confirms PEG-CLP-RGD motif might affect glioblastoma cell behavior related to the invasiveness of this cancer. The size of response of C6 glioma cells was half of that of glioblastoma HROG36 cell line. However, C6 cells were equally responsive to both RGD and IKVAV motifs. IKVAV is shown to induce glioblastoma apoptosis by immobilizing β1-integrin at the cell membrane, activating integrin-linked kinase and inhibiting focal adhesion kinase (Srikanth et al., 2013.). On the other hand, micropatterned blood vessel wall mimicking laminin stimulated migration of both rat C6 glioma cells and human glioma–propagating cells (hGPCs) isolated from a patient via Arp 2/3 and formins (Monzo et al., 2016). Thus, the exact signaling role of laminin and its active sequence in the development of glioma cell invasiveness is yet to be discovered.

Human melanoma cells A375 demonstrated higher focal adhesion number on both PEG-CLP and PEG-CLP-RGD hydrogels compared to PEG-CLP-IKVAV, and higher proliferation on PEG-CLP-RGD. This is in accordance with the recent evidence that fibronectin promotes melanoma proliferation and metastasis by inhibiting apoptosis and regulating epithelial-mesenchymal transition (Li et al., 2019). Interestingly, the changes in focal adhesion size and number per cell in A375 samples was not reflected in the spread area of individual cells. This has to be considered together with the finding that migration of A375 was not so high compared to that of glioma cells. The more prominent response of A375 to different hydrogels was proliferation, thus, the changes in adhesion pattern were mostly related to redistribution of the cytoskeleton during mitosis. This could at least partially explain why the redistribution of the focal adhesions in the cells were not so visible on their projected area. However, adhesion changes correlated well with the changes in cell shape index, indicating that the cells did respond to the contact modulation. Introduction of IKVAV sequence significantly suppressed cell migration and focal adhesion formation, and had no effect on proliferation. This is in contrast to the results obtained by Chung et al., 2011, that extrinsic laminin-332 produced by keratinocytes promotes both adhesion and migration of melanocytes and A375 melanoma cells. Although the authors have tested that soluble factors produced by keratinocytes are not enough to stimulate such migration efficiency, there still might be a possibility of simultaneous action of factors other than laminin. In general, the data about the influence of laminins and their active sequences to melanoma cell migration are contradictory and requires more detailed studies.

Phelps et al. (2012), have introduced 8- and 4-armed PEG-maleimide-hydrogels as a versatile tool for protein and cell delivery. PEG itself is a bioinert material that can form polymer chains with a defined chemical structure and have end groups that enable cross-linking. Conjugation of CLP to PEG imparts the close proximity between the CLP peptides facilitating the self-assembly, stability, and mechanical strength of the synthetized material (Islam et al., 2016). PEG-RGDS and PEG-PQ-PEG peptide hydrogel was successfully applied to model metastasis of lung adenocarcinoma (Gill et al., 2012). MMP-degradable (KGGGPQG↓IWGQERCG) crosslinking density regulating peptide and non-degradable (KGGGDQGIAGFERCG) PEG-peptide mixed together with incorporated RGD motif containing peptide (QEQVSPL-GRGDSPG) were used to encapsulate drug resistant ovarian cell lines V-MZ-6 and SKOV-3 (Loessner et al., 2010). CRGDS and a matrix metalloproteinase (MMP)-cleavable peptide were chemically incorporated and mixed with hyaluronic acid to make a brain ECM-mimicking environment to study the effects of environmental stiffness on human glioblastoma U87 cell behavior (C. Wang et al., 2014). Also, PEG-CRGDS peptides crosslinked by MMP-controlled peptide chains were designed to investigate the influence of biochemical and biophysical matrix properties on human fibrosarcoma HT-1080 cell migration. The findings of these studies support the importance of the development of hydrogel systems with defined ECM-mimicking cues as a tool for cancer biology research.

Hydrogel systems with encapsulated cells become more and more popular for *in vitro* studies because of their more realistic environmental cues. However, with the advantages come some challenges. For example, the experimental setup is more complicated and time consuming because of hydrogel preparation and the cell encapsulation procedure, the standardization and repeatability of the preparations makes it difficult to compare results provided by different research groups. There is a restriction to some evaluation assays adapted to optically transparent and monolayer cell cultures, and they are not suitable for hydrogel encapsulated cell cultures. In addition, most popular commercially available hydrogel matrices such as Matrigel^®^, Gelltrex^TM^, and others are cell derived and have undefined and batch-to-batch variable composition. They provide similar to a real tissue environment, but can hardly serve as tools for the examination of what from the environment is signaling to the cells. Taking this into account, in this study, we have decided to apply a crosslinked PEG-CLP hydrogel system that shares some similarities with conventional 2D cell culture methods, such as a smooth horizontal plane and transparency (transmission 92.4 ± 0.95%, backscatter 0.90 ± 0.17%) (Islam et al., 2016), thus allowing for sample monitoring and analysis by all conventional optical assays. Although the hydrogels do not provide spatial distribution of cells in vertical axis, they have an ECM-mimicking surface structure and elastomechanical and chemical properties, making it a possible alternative for *in vitro* cell culture models.

In conclusion, PEG-CLP hydrogels with functional cell adhesion peptide motifs RGD and IKVAV promoted different cell adhesion and morphology behavior that correlated with changes in biological activity in three cancer cell lines. Such hydrogel systems can serve as convenient and informative research models to study the role of environmental factors in tumor invasiveness.

## Data Availability Statement

The datasets generated for this study are available on request to the corresponding author.

## Author Contributions

JP designed the peptides sequences and hydrogel synthesis protocol. AV performed the synthesis of peptides and hydrogels. AJ conceived and supervised the cell culture study. ES performed the experiments on HROG36 cells. GS performed the experiments on A375 cells. IS and RB performed the experiments on C6 cells. AM-G performed immunocytochemistry experiments. ZB carried out the confocal imaging and focal adhesion evaluation. All authors participated in the manuscript preparation.

## Conflict of Interest

The CLP hydrogel matrix technology described in this manuscript is disclosed in Ferentis UAB patent applications GB1506316.7, WO2016/165788. AM-G and AV were employed by the company Ferentis UAB. The remaining authors declare that the research was conducted in the absence of any commercial or financial relationships that could be construed as a potential conflict of interest. The handling editor declared a past co-authorship with one of the authors AJ.
